# Sex-dependent dopaminergic vulnerability may contribute to sex differences in Parkinson’s disease risk

**DOI:** 10.1093/braincomms/fcag310

**Published:** 2026-08-12

**Authors:** Tamir Eisenstein, Frederik J Lange, Michele T M Hu, Johannes C Klein

**Affiliations:** Oxford Centre for Integrative Neuroimaging, FMRIB, Nuffield Department of Clinical Neurosciences, University of Oxford, Oxford OX3 9DU, UK; Oxford Parkinson’s Disease Centre, Division of Neurology, Nuffield Department of Clinical Neurosciences, University of Oxford, Oxford OX3 9DU, UK; Oxford Centre for Integrative Neuroimaging, FMRIB, Nuffield Department of Clinical Neurosciences, University of Oxford, Oxford OX3 9DU, UK; Oxford Parkinson’s Disease Centre, Division of Neurology, Nuffield Department of Clinical Neurosciences, University of Oxford, Oxford OX3 9DU, UK; Oxford Centre for Integrative Neuroimaging, FMRIB, Nuffield Department of Clinical Neurosciences, University of Oxford, Oxford OX3 9DU, UK; Oxford Parkinson’s Disease Centre, Division of Neurology, Nuffield Department of Clinical Neurosciences, University of Oxford, Oxford OX3 9DU, UK

**Keywords:** substantia nigra, nigrosome-1, Parkinson's disease, ageing, quantitative susceptibility imaging

## Abstract

Men are more likely than women to develop Parkinson’s disease, yet the biological basis of this sex difference in Parkinson’s disease risk remains unresolved. The nigrosome-1 (NG-1), a dopaminergic subregion of the substantia nigra, is selectively vulnerable to degeneration and iron accumulation in Parkinson’s disease, therefore representing a plausible locus for sex-linked vulnerability. Using iron-sensitive magnetic resonance imaging from the UK Biobank, we examined sex differences in NG-1 magnetic susceptibility across adulthood. We analysed cross-sectional data from 53 792 individuals aged 45–85 years and longitudinal follow-up in 4068 participants. Across mid-to-late adulthood, men consistently exhibited higher NG-1 magnetic susceptibility than women, suggestive of greater iron-related signal burden. In contrast, age-related trajectories and rates of within-person change were similar between sexes, with no evidence for accelerated nigrostriatal ageing in men. Sensitivity analyses showed that these sex differences were not substantially modified by genetic risk for Parkinson’s disease, cardiometabolic disease or major lifestyle factors. Together, these findings suggest that sex differences in NG-1 susceptibility are expressed primarily as a stable baseline offset rather than divergent ageing dynamics. By distinguishing baseline variation from age-related changes in a selectively vulnerable dopaminergic system, this study provides population-scale evidence relevant to understanding sex differences in vulnerability to Parkinson’s disease.

## Teaser

Eisenstein *et al*. report that men exhibit higher nigrosome-1 magnetic susceptibility than women across mid-to-late adulthood, using data from 53 000 participants. Sex differences were not explained by divergent ageing trajectories or genetic, lifestyle or cardiometabolic factors, suggesting an intrinsic male-biased dopaminergic vulnerability that may contribute to males’ higher Parkinson’s risk.

## Graphical Abstract

**Graphical Abstract:**
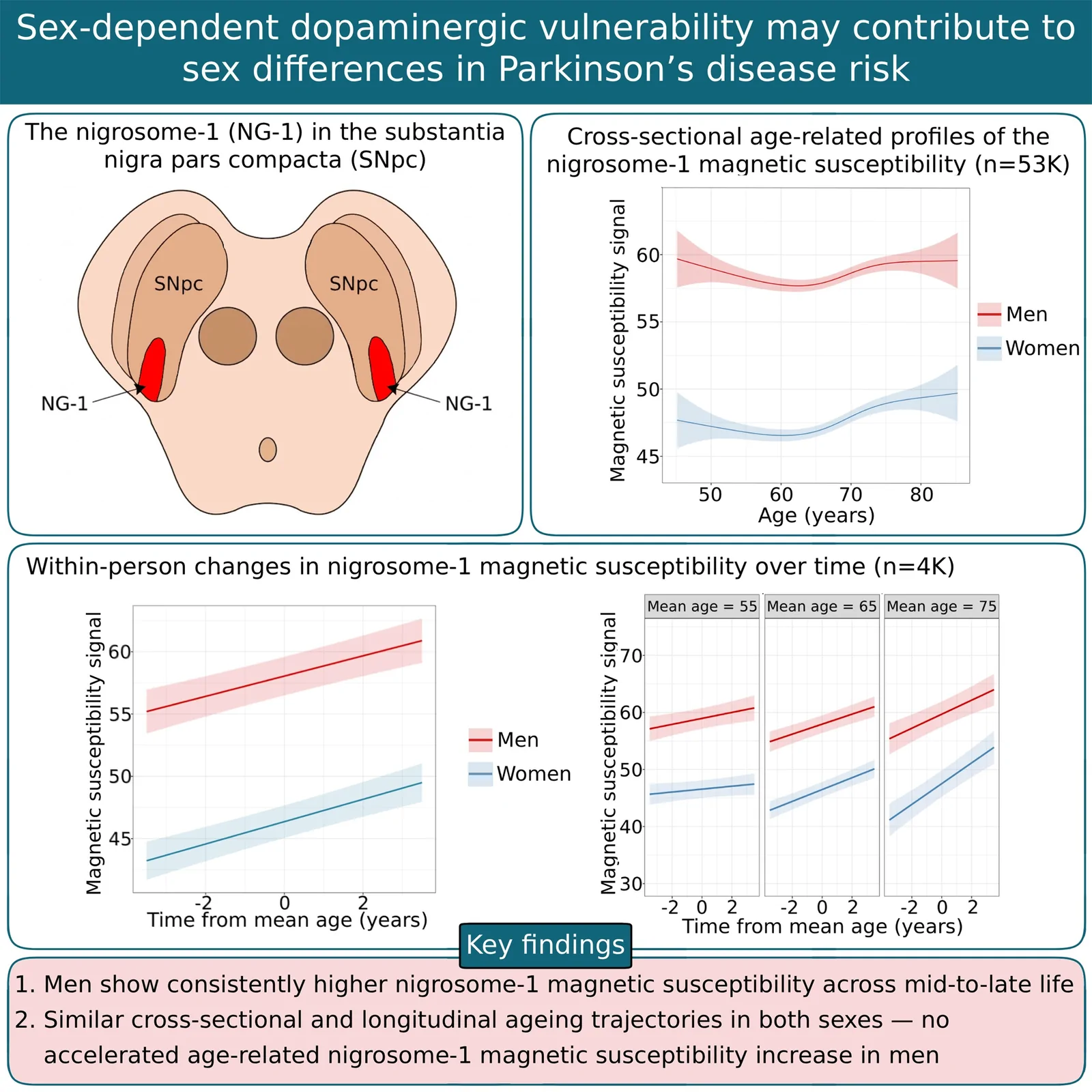

## Introduction

**Figure 1:**
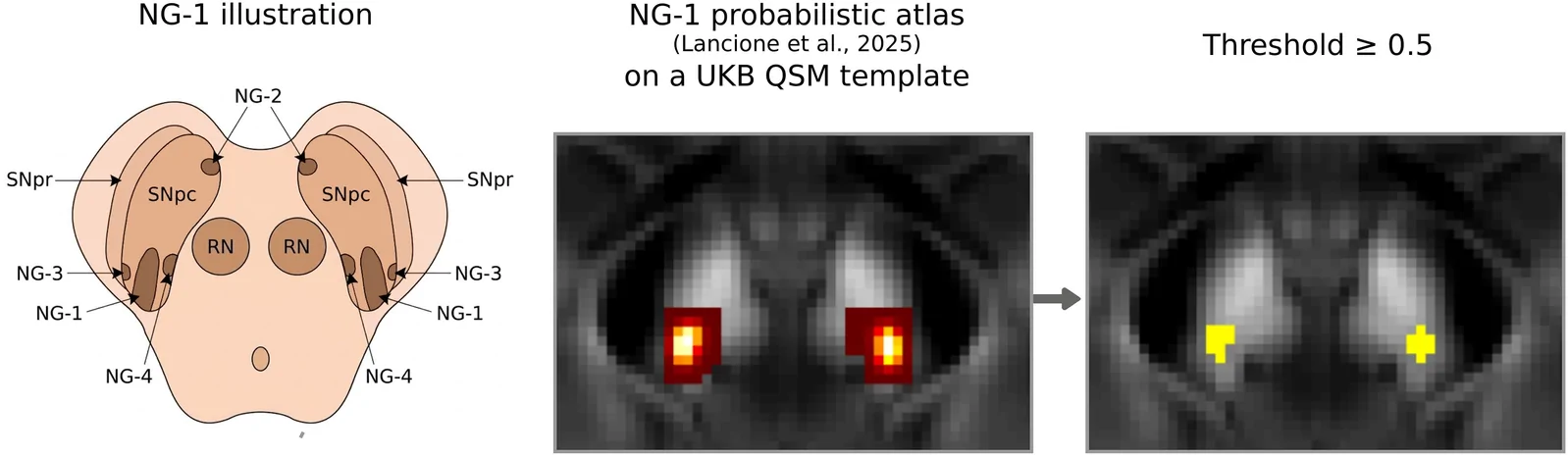
**NG-1 localization.** A schematic illustration of a transverse section through the midbrain at the level of the nigrosome-1 based on previous post-mortem studies,^7,33^ illustrating the spatial organization of the substantia nigra pars compacta (SNpc) and the positions of nigrosomes 1–4. Because the illustration depicts a single axial plane at the level of NG-1, nigrosome-5 is not shown (left panel). Bilateral masks derived from a multi-parametric *in vivo* probabilistic atlas (Lancione et al. 2025) (centre and right panels). NG, nigrosome; QSM, quantitative susceptibility mapping; RN, red nucleus; SNpc, substantia nigra pars compacta; SNpr, substantia nigra pars reticulata; UKB, UK Biobank.

Parkinson’s disease is the fastest-growing and second-most prevalent neurodegenerative disease.^1,2^ The prevalence and incidence of Parkinson’s disease are higher in men than in women, and sex differences in the onset, expression and progression of the disease have been reported.^1,3^ Yet, the biological or environmental origins of this sex difference remain unresolved. The hallmark neuropathological features of Parkinson’s disease are the loss of dopaminergic cells and increased iron deposition in the substantia nigra pars compacta (SNpc), leading to striatal dopamine depletion.^4-6^ The SN comprises five clusters of dopaminergic neurons known as nigrosomes,^7^ which exhibit a stereotyped temporospatial pattern of degeneration in Parkinson’s disease.^8^ Among these, the nigrosome-1 (NG-1) is the most vulnerable and earliest to be affected in the course of the disease. The nigrosomes are enriched in neuromelanin-containing dopaminergic neurons, in which iron is physiologically chelated. In healthy individuals, the neuromelanin-associated iron contributes to characteristic magnetic susceptibility properties that can be visualized using iron-sensitive MRI techniques such as quantitative susceptibility mapping (QSM).^6^ This enables the identification of the NG-1, which appears as a distinct hypointense subregion within the dorsolateral SNc.^9,10^ In Parkinson’s disease, degeneration of dopaminergic neurons and loss of neuromelanin lead to iron release and oxidation and increased magnetic susceptibility and QSM signal within the NG-1.^5,11,12^ Previous studies have used iron-sensitive MRI such as QSM, as a surrogate marker for increased iron deposition and dopaminergic cell loss,^13,14^ to demonstrate SNpc vulnerability in Parkinson’s disease.^9,11^ However, previous studies examining nigrostriatal sex differences in the healthy population using biochemical and MRI methods provided inconsistent findings.^15-20^ Hence, whether men and women differ with respect to innate NG-1 properties and vulnerability remains unclear. By leveraging cross-sectional and longitudinal QSM MRI data from the UK Biobank (UKB), we aimed to address the following key questions: (i) whether sex differences exist in baseline NG-1 QSM-derived magnetic susceptibility (Fig. 1), as a proxy for iron content?;^21^ (ii) do the age-related trajectories of NG-1 susceptibility differ between the sexes?; (iii) do men and women differ in NG-1 susceptibility change over time and, if so, whether such differences are age-dependent?; and (iv) whether these sex differences persist after accounting for genetic, lifestyle and cardiometabolic factors?

## Materials and methods

This study used data from the UKB as part of Data Access Application 95506. UKB has approval from the North-West Multi-Centre Research Ethics Committee to obtain and disseminate data and samples from the participants (http://www.ukbiobank.ac.uk/ethics/), and these ethical regulations cover the work in this study. Written informed consent was obtained from all participants.

### Sample characteristics

We examined 53 792 participants with baseline QSM data, aged 45–85 (53.4% females), free from major neurodegenerative diseases including Parkinson’s disease or other Parkinsonian syndromes, all-cause dementia, multiple sclerosis and cerebrovascular disease or stroke. Of these, 4068 (2181 women, 1887 men) had a follow-up MRI scan with a mean interval of 2.6 ± 1.03 years (women: 2.6 ± 1.04, range 1–7 years; men: 2.5 ± 1.01, range 1–6.75 years). The health-related status of the participants for exclusion was obtained from a combination of UK Biobank’s linked primary care data, hospital inpatient admissions, death records and self-reported medical conditions, mapped to the International Classification of Diseases (ICD-10) coding system (data category 1712), following the approach recommended by the UK Biobank.^22^ Participants were excluded if they had diagnoses or self-reported major neurodegenerative disorder/cerebrovascular disease (see Supplementary material for specific diagnoses codes).

### QSM data processing

Susceptibility-weighted imaging (SWI) scans were acquired at four UKB sites using identical 3T Siemens Skyra MRI scanners. SWI data were acquired using a three-dimensional (3D) dual-echo gradient echo sequence. T1-weighted structural images were used to align participants into the standard space atlas, and T2 fluid-attenuated inversion recovery (FLAIR) images were used to generate ventricle masks used in QSM referencing (see Supplementary material for all scanning parameters). The quality control (QC) pipeline developed for UKB brain imaging was applied to all individuals included in this study.^23^ The QSM data pre-processing consisted of several steps as previously described in detail.^24^ To optimize image registration to standard space, we used the recently developed non-linear image registration tool FSL-MMORF.^25^ The average χ values were then extracted from both the right and left NG-1 for each participant, using masks derived from a recently published high-resolution multi-parametric probabilistic *in vivo* atlas,^26^ with a threshold of 50% to adhere to visual detection of the region on healthy QSM maps. To minimize the effect of technical and motion-related confounds and harmonize the QSM data across the UKB assessment centres, we implemented a linear mixed-modelling deconfounding procedure (see Supplementary material for more details). Given minor differences between the bilateral values (see Supplementary material for direct comparison), the deconfounded NG-1 QSM values were then averaged across the right and left NG-1, and this mean value was further used as the input to the main downstream analyses.

### Statistical analysis

Statistical analyses were performed in R (v4.4). We first modelled cross-sectional age trajectories of nigrosome-1 QSM values using generalized additive models (GAMs) using the mgcv package in R, to allow for the modelling of non-linear effects of age on the NG-1 QSM signal. Therefore, age was modelled as a smooth term and fitted using cubic regression splines with a maximum of 20 basis functions. Sex was modelled as a binary fixed effect to examine mean sex differences in NG-1 QSM signal, and an age × sex smooth-factor interaction term was added to the model to examine sex differences in the shape of the age–NG1 signal relationship. UKB assessment centre was modelled as a random intercept. To evaluate potential modification effects on the relationship between sex and NG-1 signal, we ran sensitivity analyses while including an interaction term between sex and one of the following predictors: Parkinson’s disease polygenic risk score, family history of Parkinson’s disease, smoking status, alcohol consumption frequency, education level, as well as cardiovascular disease and diabetes (see Supplementary material for all relevant data fields and diagnosis codes).

The longitudinal data analysis was conducted using generalized additive mixed models (GAMM) via the gamm4 package in R. In order to separate cross-sectional and longitudinal effects of age, we decomposed age into: (i) *mean age*, representing the cross-sectional effect of age by calculating the average age across time points for each participant, and (2) *time*, representing the longitudinal effect of age by calculating the deviation of each participant's age at a given time point from their mean age.^27^ To examine sex differences in the rate of NG-1 signal change, we tested the interaction between time and sex. To examine whether sex differences vary with age, we examined the three-way interaction between time, sex and mean age. Lastly, the same sensitivity analyses were conducted for the longitudinal analysis by testing the time × sex × predictor interactions. The *P*-values of the sex × predictor interaction terms in the cross-sectional analysis and the time × sex × predictor terms in the longitudinal sensitivity analysis that are reported in the main text were corrected for multiple testing using the Benjamini–Hochberg false discovery rate (FDR).^28^ See Supplementary material for more details. All analyses used complete-case data and no imputation was performed.

## Results

### Population-based NG-1 trajectories

**Figure 2:**
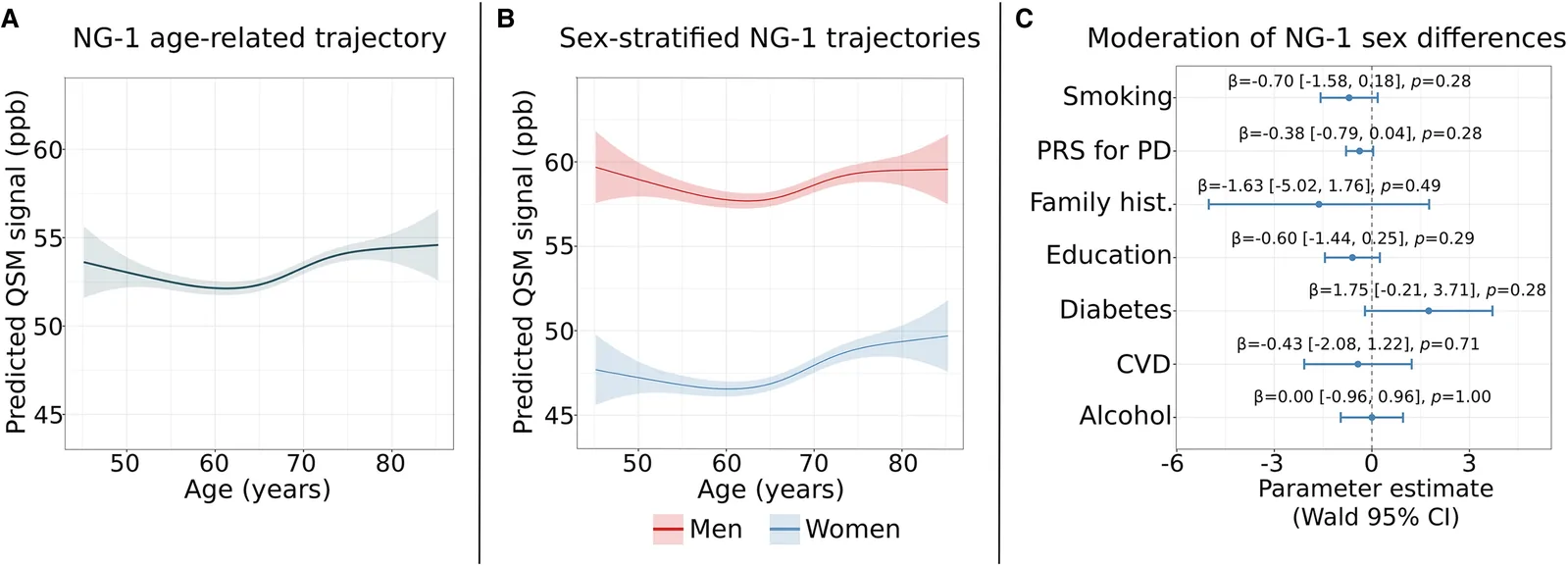
**Cross-sectional age-related trajectories of the NG-1 QSM signal during mid- to late-life.** (**A**) Generalized additive models (GAM, *n* = 53 792) were used to examine age-related trajectories of the NG-1 QSM signal across the entire sample. We identified a non-linear age-related trajectory of the NG-1 QSM signal with an increase during the seventh decade of life [effective degrees of freedom (edf) = 3.43, *P* < 0.001]. (**B**) Age-related NG-1 trajectories in men (red) and women (cyan). GAM analysis further revealed that men exhibit consistently higher NG-1 QSM across the cohort age range [β = 10.93 ppb (95% CI 10.50, 11.35), *P* < 0.001). (**C**) Sensitivity analyses incorporating linear interaction terms in the GAM models did not find effect modification of sex differences in NG-1 signal values by genetic, lifestyle or cardiometabolic factors (statistical values are presented in the figure; the sample size for each individual sensitivity model ranged between 52 269–53 792 depending on available participant data). Shaded ribbons/error bars represent 95% confidence intervals. CI, confidence interval; CVD, cardiovascular disease; NG-1, nigrosome-1; PD, Parkinson’s disease; ppb, parts per billion; PRS, polygenic risk score; QSM, quantitative susceptibility mapping.

Using generalized additive models on the full cross-sectional sample (*n* = 53 792), we found a non-linear age-related trajectory of the NG-1 QSM signal, showing a mild decline in early midlife, followed by an increase during the seventh decade of life, and further moderation towards the middle of the eighth decade [effective degrees of freedom (edf) = 3.43, *P* < 0.001] (Fig. 2A). A follow-up analysis in a subsample restricted to participants aged 45–60 (*n* = 14 456) showed that the apparent mild decline in that age range was not statistically significant on its own (edf = 1.00, *P* = 0.09), with no statistical evidence for sex differences in the shape of the trajectory (*P* = 0.61), reflecting relatively stable NG-1 QSM values across this age range. Men demonstrated a consistently higher NG-1 QSM signal across the age range of the sample, from mid- to late-adulthood [β = 10.93 (95% CI 10.50, 11.35), *P* < 0.001], with no statistical evidence for sex difference in the shape of the age-related trajectory (*P* = 0.06) (Fig. 2B). Sensitivity analyses showed no consistent modification of these baseline sex differences by either genetic or family risk, lifestyle factors or cardiometabolic disease (*pFDR* > 0.28, Fig. 2C).

### Longitudinal changes in NG-1

**Figure 3:**
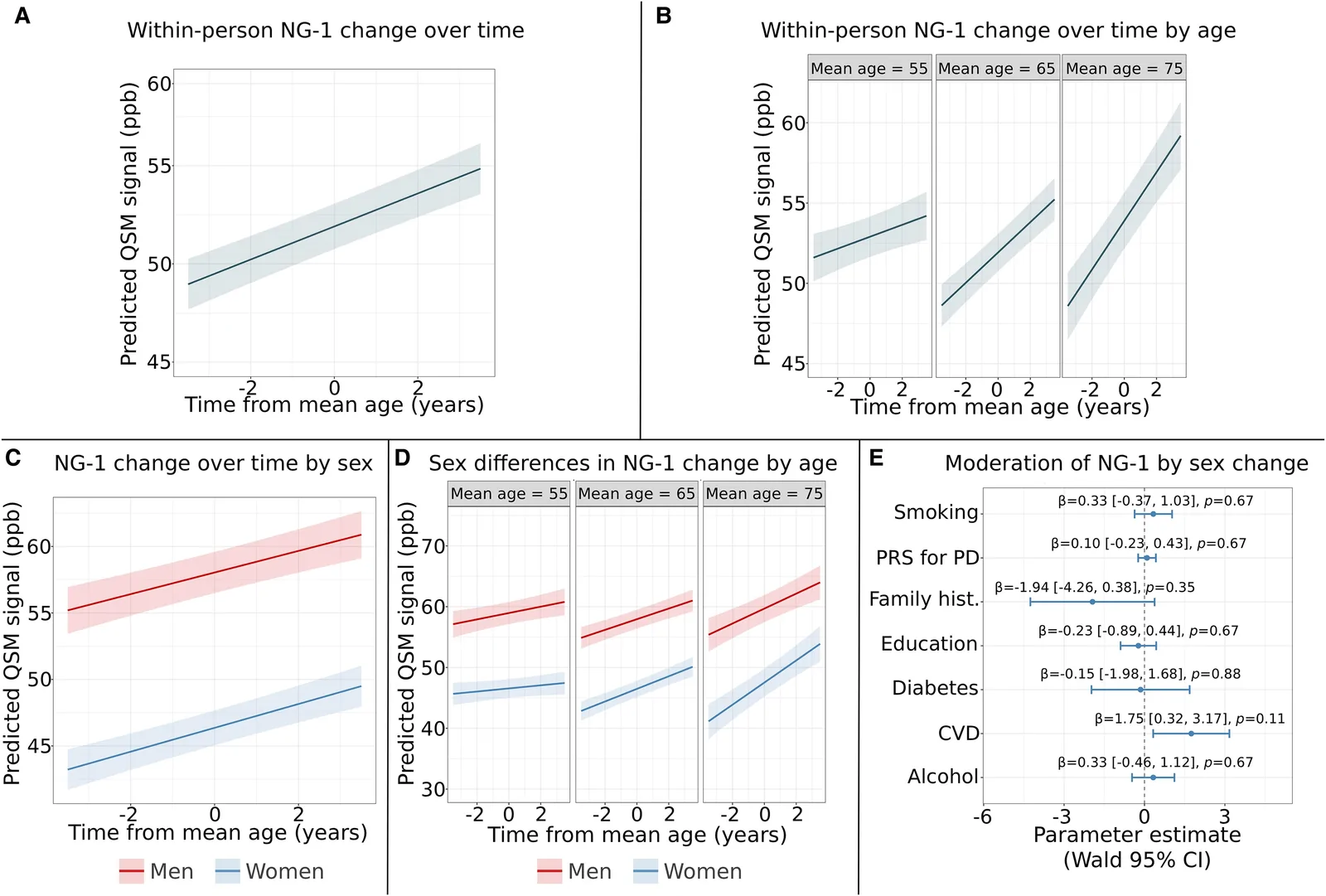
**Within-person changes in NG-1 QSM signal over time.** Longitudinal changes in NG-1 signal were examined using generalized additive mixed models (GAMM, *n* = 4068). (**A**) Within-person increase over time in NG-1 QSM signal was found across the entire longitudinal sub-sample [β = 0.84/year (95% CI 0.67, 1.01), *P* < 0.001]. (**B**) The rate of increase was further found to be strongly moderated by age, with older age associated with the higher rate of NG-1 signal increase [time-interval × age interaction: β = 0.06/year per year of age (95% CI 0.04, 0.08), *P* < 0.001]. (**C**) Within-person changes over time in NG-1 signal did not differ between men (red) and women (cyan) [time × sex: β = −0.08 (95% CI −0.42, 0.25), *P* = 0.627]. (**D**) Age did not moderate sex differences in within-person rate of NG-1 change over time [time × sex × age interaction: β = −0.04 (95% CI −0.09, 0.003), *P* = 0.064]. (**E**) No modification of sex differences in NG-1 signal change over time by genetic, lifestyle or cardiometabolic factors were observed (statistical values are presented in the figure; the sample size for each individual sensitivity model ranged between 3960–4068 depending on available participants data). Shaded ribbons/error bars represent 95% confidence intervals. CI, confidence interval; CVD, cardiovascular disease; NG-1, nigrosome-1; PD, Parkinson’s disease; ppb, parts per billion; PRS, polygenic risk score; QSM, quantitative susceptibility mapping.

We next ran generalized additive mixed models to first examine how the NG-1 QSM signal behaves when measured over time within individuals. Within-person NG-1 signal increased over time [β = 0.84/year (95% CI 0.67, 1.01), *P* < 0.001] (Fig. 3A), and this rate of increase was strongly moderated by age with older adults exhibiting steeper signal increase over time [time-interval × age: β = 0.06/year per year of age (95% CI 0.04, 0.08), *P* < 0.001] (Fig. 3B). However, the rate of within-person NG-1 increase did not differ between sexes [time × sex: β = −0.08 (95% CI −0.42, 0.25), *P* = 0.627] (Fig. 3C), nor did we find evidence that sex differences in NG-1 signal change varied with age [time × sex × age: β = −0.04 (95% CI −0.09, 0.003), *P* = 0.064] (Fig. 3D). Sensitivity analyses did not show consistent effect modification (*p*FDR > 0.11), with the partial exception of cardiovascular disease which, while associated with a modestly larger male–female difference in NG-1 signal change over time (β = 1.75 [95% CI 0.32, 3.17]), did not survive multiple testing correction (Fig. 3E).

## Discussion

In this large population-based study, we demonstrate that sex differences in NG-1 magnetic susceptibility are expressed primarily as a stable baseline difference across mid-to-late adulthood, rather than as divergent ageing trajectories. Men consistently exhibited higher NG-1 susceptibility than women, while age-related trajectories and rates of within-person change were comparable between sexes. These findings in turn argue against accelerated nigrostriatal ageing in men and instead support a model of an intrinsic male-biased vulnerability of the NG-1 in which sex differences in nigral biology are established prior to midlife and persist into ageing.

The NG-1 is the most selectively vulnerable dopaminergic subregion of the substantia nigra in Parkinson’s disease,^8^ and increased iron-related susceptibility in this region aligns with known pathological patterns observed in the disease.^13^ By examining NG-1 biology in the absence of overt neurodegenerative disease, our findings isolate baseline variation from disease-related changes and provide population-scale context for interpreting sex differences in Parkinson’s disease risk. Importantly, the directionality and anatomical specificity of the observed sex difference are consistent with established markers of nigral vulnerability in Parkinson’s disease.^29^

Prior studies have yielded inconsistent results regarding sex differences in the nigrostriatal system, which could arise from heterogeneous methodology (e.g. different *in vivo* MRI metrics, post-mortem biochemistry), small samples and cross-sectional designs.^15-20^. For example, while Xu *et al*. (2008) did not find sex differences in the SN using susceptibility-weighted imaging (SWI),^19^ Persson *et al.* (2015) found lower QSM values in women when accounting for age.^20^

By leveraging large-scale cross-sectional and longitudinal data, the present study clarifies that sex differences in NG-1 susceptibility are not driven by differences in ageing dynamics. This is consistent with broader evidence for biological differences between the sexes, which may explain differences in resilience and risk to the ageing process.^30^ Sensitivity analyses further indicated that these baseline differences were not substantially modified by genetic risk for Parkinson’s disease, family history, lifestyle factors or cardiometabolic disease, supporting the interpretation of a stable biological difference rather than secondary modulation.

It should be noted that the observed cross-sectional age-related trajectories of the NG-1 QSM values did not mirror the observed patterns in the longitudinal data, in which increased age was associated with a greater rate of increase. In accordance with this observation, it was recently shown that inferring age-related trajectories of brain changes from cross-sectional data may underestimate brain changes that are directly measured with longitudinal data.^31^ Moreover, it was found that such divergence may vary with age and brain metric examined. Additional large-scale prospective studies will be valuable in confirming the within-person trajectories of the NG-1 and improving their utilization for individual and prospective predictions. However, it should also be noted that the stable sex differences persisted regardless of the nature of the data used to model the age-related changes in NG-1 signal in the current study, supporting this feature as a consistent biological phenomenon.

### Limitations

Several limitations should be considered. First, the UKB cohort is comprised of a predominantly white ethnic background, limiting generalizability to other populations. In addition, the longitudinal follow-up interval was relatively short, and younger age ranges are not available in the UKB, precluding direct assessment of when these sex differences emerge earlier in life. It should be noted that by excluding individuals with neurodegenerative and cerebrovascular diagnoses, the present sample represents a population that is largely free from significant neurological insults that are associated with older age. While the low global prevalence of Parkinson’s disease in people over the age of 60 years (∼1%)^32^ means that this exclusion is unlikely to produce meaningful survivor bias at the population level, interpreting NG-1 susceptibility as a vulnerability marker in individuals who have not developed Parkinson’s disease should be interpreted with caution. An elevated NG-1 susceptibility in men may reflect a necessary but insufficient condition for the disease, consistent with a multifactorial model in which additional genetic and environmental factors also play key roles in whether increased nigral vulnerability ultimately translates to clinical Parkinson’s disease. The oldest men in the cohort, who have maintained elevated NG-1 susceptibility without developing the disease, may therefore include resilient individuals or those lacking additional necessary risk factors.

## Conclusions

Our findings support a male-biased NG-1 biological vulnerability, which may contribute to their higher lifetime prevalence of Parkinson’s disease and help explain the male sex as a Parkinson’s disease risk factor. The consistency of this difference across mid- to later adulthood, and its independence from major genetic and lifestyle factors, positions the NG-1 as a potential biological link connecting sex to Parkinson’s disease risk. Elucidating the genetic and molecular mechanisms that establish this baseline offset in NG-1 biology, and how they may interact with modifiable exposures, could ultimately inform targeted prevention or neuroprotective interventions aimed at reducing Parkinson’s disease risk in vulnerable populations. Whether interventions targeting iron metabolism in midlife could delay or prevent Parkinson’s disease onset remains an important question for future research.

## Supplementary Material

fcag310_Supplementary_Data

## Data Availability

UK Biobank data are available for approved researchers through a procedure described at https://www.ukbiobank.ac.uk/enable-your-research. Permission to use the UKB Resource was obtained as part of Data Access Application 95506. The R code supporting the analyses in this paper is available at https://github.com/TamirEisenstein/NG1_by_sex
