# Supplementary material for: Sex-dependent dopaminergic vulnerability may contribute to sex differences in Parkinson’s disease risk
Source: Brain Commun. 2026 Aug 12;8(4):fcag310. doi: 10.1093/braincomms/fcag310 (PMC13519322; doi:10.1093/braincomms/fcag310)
Supplement: Supplementary file 1 [file fcag310_supplementary_data.docx]

**Sex-dependent dopaminergic vulnerability may contribute to sex differences in Parkinson’s disease risk**

**Supplementary Information**

**Participants**

The health-related status of the participants for exclusion and for the identification of cardiovascular/diabetes cases was obtained from a combination of UK Biobank’s linked primary care data, hospital inpatient admissions, death records and self-reported medical conditions, mapped to the International Classification of Diseases (ICD-10) coding system (data category 1712), following the approach recommended by the UK Biobank (1). Participants were excluded if they had diagnoses or self-report of major neurodegenerative disorder/cerebrovascular disease, based on the following UK Biobank *First Occurrence* codes F01, F02, F03, F04, G30, G20, G21, G22, G23, G25, G20, G21, G22, G23, G25, I60, I61, I62, I63, I64, I65, I66, I67, I68, I69, and *Self-Report* codes: 1081, 1086, 1261, 1262, 1263, 1491, 1583.

**QSM data processing**

**Data acquisition**

The acquisition protocol of the UK Biobank 3-Tesla QSM imaging can be found here: <https://biobank.ctsu.ox.ac.uk/crystal/crystal/docs/brain_mri.pdf>, and a detailed overview of the brain MRI acquisition protocols used in UK Biobank has been previously described (2). Susceptibility-weighted imaging (SWI) scans were acquired at four sites using identical 3T Siemens Skyra MRI scanners (software platform VD13) with 32-channel head receive coils. SWI data were acquired using a three-dimensional (3D) dual-echo gradient echo sequence with the following parameters: voxel size = 0.8 × 0.8 × 3 mm^3^, matrix size = 256 × 288 × 48 (whole-brain coverage), echo times (TE1 /TE2 ) = 9.4/20 ms, repetition time (TR) = 27 ms, in-plane acceleration = 2 and total scan time = 2:34 minutes. Magnitude and phase data from each receive channel were saved for offline coil combination, as described below. T1-weighted structural images were used to align participants into the standard space atlas and T2 fluid attenuated inversion recovery (FLAIR) images were used to generate ventricle masks used in QSM referencing. T1-weighted images were acquired with 3D MPRAGE, voxel size = 1 × 1 × 1 mm^3^, matrix size = 208 × 256 × 256, inversion time (TI)/TR = 880/2,000 ms, in-plane acceleration = 2, total scan time = 4:54 minutes. The T2-weighted FLAIR scans were acquired with 3D SPACE, voxel size = 1.05 × 1 × 1 mm^3^, matrix size = 192 × 256 × 56, TI/ TR = 1,800/5,000 ms, in-plane acceleration = 2, total scan time = 5:52 minutes. The quality control (QC) pipeline developed for UK Biobank brain imaging was applied to all individuals included in this study (3).

**Data processing pipeline**

The QSM data preprocessing consisted of several steps as previously described in details (4). Briefly, individual channel phase images for each echo were combined using MCPC 3D-S and unwrapped using a Laplacian-based algorithm, and the two echoes were combined with weighted averaging. Brain-edge voxels with extremely large phase variance (primarily near sinuses) were detected and excluded. Background fields were removed using the variable kernel sophisticated harmonic artifact reduction for phase data (V-SHARP) algorithm. Next, maps of the quantitative estimates of the shifted magnetic susceptibility (χ maps) were calculated using iLSQR21 and referenced to cerebrospinal fluid (CSF) in the lateral ventricles. To optimize image registration to standard space, we used the recently developed nonlinear image registration tool FSL-MMORF, which provides an optimised registration framework that leverages rich information from multiple scalar and tensor image modalities such as structural and diffusion images (5). The average χ values were then extracted from both the right and left NG-1 for each participant, using right and left NG-1 masks derived from a recently published high resolution and multi-parametric probabilistic *in vivo* atlas (6), with a threshold of 50% to adhere with visual detection of the region on healthy QSM maps. Next, to minimize the effect of technical and motion-related confounds on the QSM values and harmonize the QSM data across the UK biobank assessment centres, we implemented a linear mixed modelling deconfounding procedure before the main downstream statistical analyses of the study (7). We first defined a set of variables representing potential confounders, namely, head-motion estimate, as well as table and head positions in the scanner (x, y, z). For each imaging confound measure, values more extreme than 15 median absolute deviations (MAD) were excluded as potential outliers as previously suggested, given that some confounders may have extremely non-Gaussian underlying distributions (8). Similarly, participants with NG-1 signal values more extreme than 8 MAD were also excluded (4, 8). After outlier removal, we used the linear mixed-effect models to regress the NG-1 values (left and right separately) on this set of imaging confounders. We included the linear as well as the squares of these variables to also model potential nonlinear effects of the imaging confound measures. UK Biobank assessment centres were modelled as a random intercept. The residuals of these analyses. i.e., the deconfounded NG-1 QSM values, were then averaged across the right and left NG-1 given minor differences between the bilateral values. We examined potential laterality effect using paired t-tests comparing the left and right NG-1 QSM values separately for the males (n=25,012) and females (n=28,702) in the sample. Although both analyses revealed statistically significant difference (**males** mean difference = -0.77 ppb; 95% CI [−1.06, -0.48]; *p* = 0.001; **females** mean difference = 0.69 ppb; 95% CI [0.44, 0.94]; *p* < 0.001), the magnitudes of these mean differences were very small (i.e., of less than 1 ppb), and are negligible relative to the between-subject variability (SD ranges between 27-28 ppb for either side in both males and females) and to the main effect of sex observed in the study, suggesting no meaningful underlying biological laterality effect, and the direct consequence of the large sample sizes.

**Statistical analysis**

Statistical analyses were performed in R (v4.4). We first modelled cross-sectional age trajectories of nigrosome-1 QSM values using generalized additive models (GAMs) using the mgcv package in R, to allow the modelling of non-linear effects of age on the NG-1 QSM signal. Therefore, age was modelled as a smooth term and wad fitted using cubic regression splines with a maximum of 20 basis functions. Sex was entered to the model as a binary fixed effect to examine mean sex differences in NG-1 QSM signal, and an age x sex smooth-factor interaction term was added to the model to examine sex differences in the shape of the age-NG1 signal relationship. UK Biobank assessment centre was modelled as a random interpret smooth. To evaluate potential confounding effects on the relationship between sex and NG-1 signal, we ran sensitivity analyses by refitted the same GAM model, each time including an interaction term between sex and one of the following predictors: PD polygenic risk score (UK Biobank field 26260, n = 52,269), family history of PD (UK Biobank field 20107, 20110, 20111, n = 53,792), smoking status (UK Biobank field 20116, n = 53,254), alcohol consumption frequency (UK Biobank field 1558, n = 53,414), higher education yes/no (based on UK Biobank field 6138, n = 53,433), cardiovascular disease (first occurrence codes I20, I21, I22, I23, I24, I25, I11, I12, I13, I50; self-report codes 1074, 1075, 1076, n = 4,093 with a diagnosis), and diabetes (first occurrence codes E11; self-report codes 1220, 1223, n = 2,766 with a diagnosis).

The longitudinal data analysis followed the work by Ravndal et al (2025) (9), and was conducted using generalized additive mixed models (GAMMs) implemented via the gamm4 package in R. In order to separate cross-sectional and longitudinal effects of age, we decomposed age into 1) ***mean-age***, representing the cross-sectional effect of age by calculating the average age across time points for each participant, and 2) ***time***, representing the longitudinal effect of age by calculating the deviation of each participant's age at a given time point from their mean-age (9). Mean-age was modelled as a smooth term with cubic regression splines with a maximum of 20 basis functions. Time was modelled as a linear term due to the relatively short within-person follow-up period. Sex was modelled as a binary factor. The models also included random intercept for UK Biobank assessment centre, and random slopes of time for each participant. To examine sex-differences in the rate of NG-1 signal change, a linear time x sex interaction term was entered to the model, as well as a linear mean-age x sex interaction term. A mean-age x sex smooth-factor was also modelled to account for non-linear cross sectional sex differences. Then, to examine whether sex differences vary with age, we fitted a model with a three-way time x sex x mean-age interaction, with all lower-order interactions including the mean-age x sex smooth-factor interaction, and the linear interaction between time and sex and mean-age and time. Lastly, the same sensitivity analyses were conducted for the longitudinal analysis by adding the following interaction terms to each model: sex x predictor, time x predictor, and time x sex x predictor. The *p*-values of the sex x predictor interaction terms in the cross-sectional analysis and the time x sex x predictor terms in the longitudinal sensitivity analysis that are reported in the main text were corrected for multiple testing using the Benjamini–Hochberg false discovery rate (FDR) (10).

**References**

1.	C. S. Kathryn Bush, Kristiina Rannikmae, Tim Wilkinson, Christian Schnier, “Definitions of Parkinson’s Disease and the Major Causes of Parkinsonism, UK Biobank Phase 1 Outcomes Adjudication” (2018).

2.	K. L. Miller, *et al.*, Multimodal population brain imaging in the UK Biobank prospective epidemiological study. *Nat. Neurosci.* **19**, 1523–1536 (2016).

3.	F. Alfaro-Almagro, *et al.*, Image processing and Quality Control for the first 10,000 brain imaging datasets from UK Biobank. *Neuroimage* **166**, 400–424 (2018).

4.	C. Wang, *et al.*, Phenotypic and genetic associations of quantitative magnetic susceptibility in UK Biobank brain imaging. *Nat. Neurosci.* **25**, 818–831 (2022).

5.	F. J. Lange, *et al.*, MMORF—FSL’s MultiMOdal Registration Framework. *Imaging Neurosci.* **2**, 1–30 (2024).

6.	M. Lancione, *et al.*, High resolution multi-parametric probabilistic in vivo atlas of dorsolateral nigral hyperintensity via 7 T MRI. *Sci. Data* **12**, 958 (2025).

7.	S. Kim, *et al.*, Harmonization of Multicenter Cortical Thickness Data by Linear Mixed Effect Model. *Front. Aging Neurosci.* **14**, 869387 (2022).

8.	G. Douaud, *et al.*, SARS-CoV-2 is associated with changes in brain structure in UK Biobank. *Nature* **604**, 697–707 (2022).

9.	A. Ravndal, *et al.*, Sex differences in healthy brain aging are unlikely to explain higher Alzheimer’s disease prevalence in women. *Proc. Natl. Acad. Sci.* **122**, e2510486122 (2025).

10.	Y. Benjamini, Y. Hochberg, Benjamini Y, Hochberg Y. Controlling the False Discovery Rate: A Practical and Powerful Approach to Multiple Testing. J. R. Stat. Soc. Ser. B. 1995:p. 289--300. *J. R. Stat. Soc. B* (1995) https:/doi.org/10.2307/2346101.
